# Electrophysiological Properties of Adult Zebrafish Oligodendrocyte Progenitor Cells

**DOI:** 10.3389/fncel.2019.00102

**Published:** 2019-04-12

**Authors:** Vasiliki Tsata, Volker Kroehne, Susanne Reinhardt, Ali El-Armouche, Michael Brand, Michael Wagner, Michell M. Reimer

**Affiliations:** ^1^Center for Regenerative Therapies TU Dresden (CRTD) and Center for Molecular and Cellular Bioengineering (CMCB), Technische Universitaet, Dresden, Germany; ^2^Dresden Genome Center, Center for Regenerative Therapies TU Dresden (CRTD), Center for Molecular and Cellular Bioengineering (CMCB), Technische Universitaet Dresden, Dresden, Germany; ^3^Department of Pharmacology and Toxicology, Technische Universitaet Dresden, Dresden, Germany; ^4^Department of Rhythmology, Heart Center Dresden, Technische Universitaet Dresden, Dresden, Germany

**Keywords:** adult zebrafish, spinal cord, oligodendrocyte progenitor cells, *in vitro*, electrophysiology, patch-clamp, ion channels, glutamate receptors

## Abstract

Low remyelination efficiency after spinal cord injury (SCI) is a major restraint to successful axonal and functional regeneration in mammals. In contrast, adult zebrafish can: (i) regenerate oligodendrocytes and myelin sheaths within 2 weeks post lesion; (ii) re-grow axonal projections across the lesion site and (iii) recover locomotor function within 6 weeks after spinal cord transection. However, little is known about the intrinsic properties of oligodendrocyte progenitor cells (OPCs), the remyelinating cells of the central nervous system (CNS). Here, we demonstrate that purified OPCs from the adult zebrafish spinal cord are electrically active. They functionally express voltage-gated K^+^ and Na^+^ channels, glutamate receptors and exhibit depolarizing, tetrodotoxin (TTX)-sensitive spikes, as previously seen in rodent and human OPCs. Furthermore, we show that the percentage of zebrafish OPCs exhibiting depolarizing spikes and Na_v_-mediated currents is lower as compared to rodent white matter OPCs, where these membrane characteristics have been shown to underlie OPC injury susceptibility. These findings imply that adult zebrafish OPCs resemble electrical properties found in mammals and represent a relevant cell type towards understanding the biology of the primary cells targeted in remyelination therapies for non-regenerative species. The *in vitro* platform introduced in this study could be used in the future to: (i) elucidate how membrane characteristics of zebrafish OPCs change upon injury and (ii) identify potential signaling components underlying OPC injury recognition.

## Main Points

– Adult zebrafish oligodendrocyte progenitor cells (OPCs) are electrically active.
– OPCs express functional voltage-gated K^+^ and Na^+^ channels.
– OPCs exhibit tetrodotoxin (TTX)-sensitive, depolarizing spikes.
– OPCs express functional glutamate receptors.

## Introduction

Oligodendrocyte progenitor cells (OPCs) give rise to myelinating oligodendrocytes that insulate, support and maintain the impulse-carrying axon, thereby contributing to proper functioning of the central nervous system (CNS). In the adult mammalian CNS, OPCs are the main proliferative population and remain highly dynamic, maintaining their population in both homeostatic and injury conditions (Ffrench-Constant and Raff, 1986; Hughes et al., 2013; Robins et al., 2013). As mature oligodendrocytes are terminally differentiated and therefore post-mitotic (Keirstead and Blakemore, 1997), only OPCs can contribute to experience- or aging-dependent myelination, throughout life (Young et al., 2013). Importantly, in cases of trauma, OPCs are the only known source of mature oligodendrocytes to allow the maintenance and survival of potentially injured or spared axons *in vivo*. Remyelination has been also shown to reverse at least part of the functional deficits persisting after injury. Both in cat and rat models of spinal cord injury (SCI), remyelination restored the conduction velocity of spared axons (Smith et al., 1979) and abolished locomotor deficits allowing functional recovery (Jeffery and Blakemore, 1997; Duncan et al., 2009). Furthermore, transplantation of myelinating progenitors remyelinated the injured spinal cord (Groves et al., 1993; Franklin et al., 1996) and improved behavioral and electrophysiological outcome in rodents (Bambakidis and Miller, 2004; Keirstead et al., 2005; Chen et al., 2014). Sontheimer et al. (1989) characterized the membrane currents of cultured murine oligodendrocytes and their progenitors, using the patch-clamp technique. The authors showed that expression of ion channels correlated with the differentiation stages of OPCs with most immature cells expressing four types of voltage-gated K^+^ and Na^+^ channels that were subsequently downregulated as OPCs progressed towards more differentiated states (Sontheimer et al., 1989). These findings suggested that excitable membrane characteristics of OPCs play an essential role to their physiological functions and could regulate their lineage progression and differentiation into mature myelinating oligodendrocytes. In 2000, in a pivotal study on cells of the oligodendroglial-lineage, using patch-clamp recordings on rat hippocampal slices, Bergles et al. (2000) showed that stimulation of excitatory axons in both young and adult hippocampus induces glutamate-mediated inward currents in OPCs that form synaptic junctions with vesicle-filled axonal terminals. The existence of such an active signaling pathway between neurons and OPCs indicated for the first time that these cells are able to sense and respond to glutamatergic input and suggested a prominent role for these cells, both in the developing and the adult mammalian CNS. To date, mammalian OPCs have been shown to form specific chemical synapses with neurons, while action potentials along axons and vesicular release in these terminals can activate both glutamate and γ-amino butyric acid (GABA) receptors (Lin and Bergles, 2004; Lin et al., 2005; Kukley et al., 2007, 2008). Interestingly, the progression of OPCs to the pre-myelinating and myelinating stage correlates with a downregulation of synaptic input (De Biase et al., 2010; Kukley et al., 2010). Given the importance of glutamatergic signaling and the glutamate-mediated damage in cases on injury in the CNS, these studies provided a new context of potential additional neuromodulatory roles of OPCs.

To date, there are no data on the electrical properties of OPCs in an adult regenerative species. Thus, we examined the functional expression of cell-type specific ion channels in OPCs from the adult zebrafish spinal cord by whole-cell patch-clamp. To access adult zebrafish OPCs, we first developed an *in vitro* system for the culture of primary OPCs from the adult zebrafish spinal cord (Kroehne et al., 2017). This setup enables the robust and highly reproducible isolation of OPCs by fluorescence activated cell sorting (FACS). Importantly, sorted OPCs remain viable in an undifferentiated state up to 10 days in culture and can be subsequently used in a variety of downstream *in vitro* approaches. In the study presented here, we elucidated OPC-intrinsic properties by whole-cell patch-clamp *in vitro*. Analysis of purified adult zebrafish OPCs demonstrates that they are electrically active, with conserved membrane properties across species. They express functional voltage-gated K^+^ and Na^+^ channels, α-amino-3-hydroxy-5-methyl-4-isoxazolepropionic acid (AMPA) receptors and exhibit tetrodotoxin (TTX)-sensitive spikes, similar to what has been previously described in human pluripotent stem cell (hPSC)-derived OPCs (Livesey et al., 2016).

Given the highly competent regenerative capacity of adult zebrafish, a thorough knowledge of the intrinsic properties of remyelinating OPCs may be a valuable asset towards the development of novel remyelination therapies for non-regenerative species.

## Materials and Methods

All experiments were performed in compliance with animal welfare legislation and were approved by the Regierungspräsidium Dresden, Germany: AZ 24-9168.24-1/2014-4 and TV 9/2016. All efforts were made to minimize animal suffering and the number of animals used.

### Fish Maintenance

Fish were kept and bred in our fish facility according to standard methods (Westerfield, 1995). We used Tg(*olig2*:eGFP) (Shin et al., 2003) and Tg(*mbp*:eGFP) zebrafish (Jung et al., 2010).

### Adult Zebrafish OPC Isolation by Fluorescence Activated Cell Sorting

Isolation and FACS of OPCs from the adult zebrafish spinal cord was adapted from Kroehne et al. (2017). Briefly, transgenic Tg(*olig2*:eGFP) adult zebrafish of 6–12 months old were terminally anesthetized in 0.1% w/vol Tricaine (Sigma Aldrich) in E3 medium for zebrafish with 10^−5^% v/v methylene blue. The skin and musculature were removed dorsally until the spinal cord was exposed and carefully removed with fine forceps. Upon dissection, up to eight spinal cords were transferred in 1 mL of Hank’s Balanced Salt Solution (HBSS; Gibco) and kept constantly on ice to avoid cell degradation. For dissociation, spinal cords were transferred with fine forceps in 100 μL of 0.25% w/vol Trypsin (Sigma; T4549) in 1 mM ethylenediaminetetraacetic acid (EDTA), incubated for 3 min at room temperature and triturated during the whole incubation using a 100 μL pipette, until full dissociation into a single cell suspension. Tissue digestion was stopped by addition of 100 μL of 20% fetal calf serum (Sigma; F0804) in 2 mM CaCl_2_ and 300 μL sterile HBSS. The cell suspension was allowed to sit on ice for 5 min and was applied to a 20 μm cell strainer (Miltenyi Biotec, # 130-101-812). After subsequent washing with 9 ml of sterile HBSS, the cells were pelleted by centrifugation at 300 *g* for 5 min at room temperature. The supernatant was discarded and the pellet was re-suspended in 1.5 ml sterile HBSS. To stain cellular nuclei, 1 μl of Vybrant^TM^ DyeCycle^TM^ Violet Stain (V35003, Molecular Probes, Invitrogen) was added to the cell suspension that was protected from light and incubated for 30 min at 28°C until cell sorting.

OPCs were sorted directly into single wells of a 96-well plate filled with culture media, using a BD FACSAria sorter. For the detection of GFP a 488 nm excitation laser and a 530/30 bandpass filter were used. Vybrant^TM^ DyeCycle^TM^ Violet Stain was detected after 405 nm excitation using a 450/40 bandpass filter (see Graphical Graphical Abstract).

**GRAPHICAL ABSTRACT:**
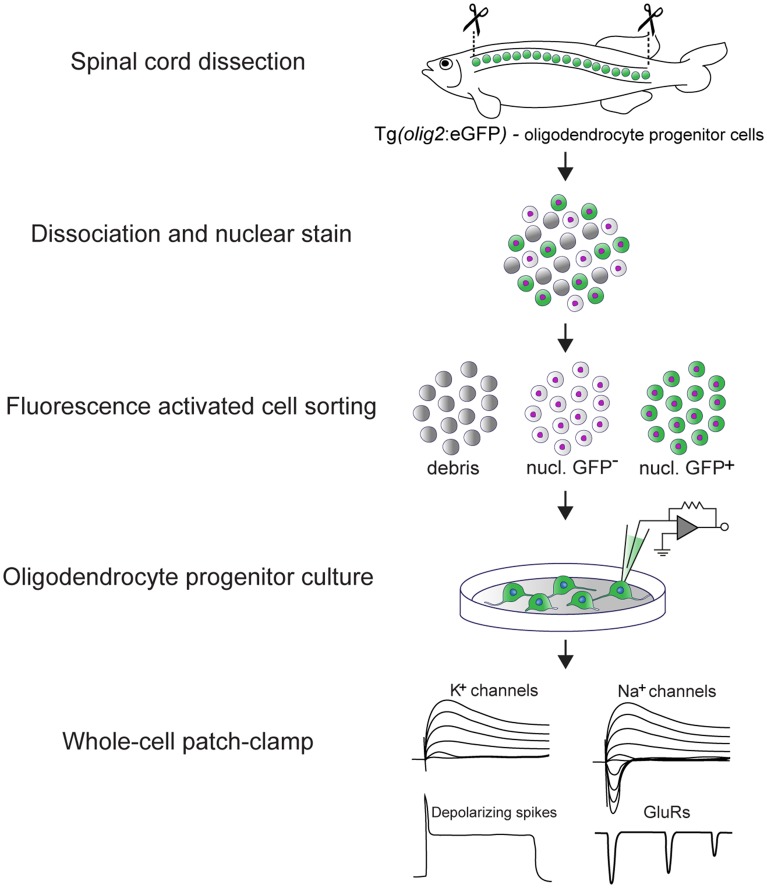
Experimental pipeline to assess the electrical properties of adult zebrafish OPCs *in vitro* and main findings. The spinal cord of transgenic Tg(*olig2*:eGFP) adult zebrafish is dissected and dissociated. In this line, eGFP is driven by the *olig2* promoter (Shin et al., 2003) that in the adult zebrafish spinal cord labels parenchymal OPCs. OPCs are subsequently purified by fluorescence activated cell sorting (FACS) and set in culture immediately after sorting. OPCs remain viable and undifferentiated for up to 10 days *in vitro* (DIV) (Kroehne et al., 2017). At 3 DIV, whole-cell patch-clamp is used to assess their electrical properties. Adult zebrafish OPCs are electrically active, they express functional voltage-gated K^+^ and Na^+^ channels, glutamate receptors and exhibit depolarizing, TTX-sensitive spikes.

### Analysis of RNA Sequencing Data

In order to support the results of the patch-clamp, a recently published RNAseq dataset of OPCs and oligodendrocytes from the adult zebrafish spinal cord from our laboratory (Kroehne et al., 2017) was investigated for genes related to ion channels. Briefly, this dataset was generated by using SMARTer Ultra Low Input RNA for Illumina Sequencing Kit—HV from Clontech, followed by a library prep using the NEBNext Ultra DNA Library Prep Kit for Illumina (NEB). The libraries were sequenced with 75 bp single end on a Hiseq2500 and mapped to GRCz10 with GSNAP (v 2016-09-23). Transcripts were counted with featureCounts v 1.5.2 based on Ensembl version 81. The raw gene read counts were converted into transcript per kilobase million (TPM) values in order to make samples comparable. Monitoring the expression of genes from diverse ion channels, the RNAseq data was filtered for gene ontology (GO) terms related to ion channels using the BiomaRt package in R analysis software.

### OPC Monocultures

The OPC monoculture strategy was adapted from Kroehne et al. (2017). Briefly, 2 days before spinal cord dissection, 5 mm round glass coverslips (Thermo Scientific) were transferred from a 70% ethanol solution to single wells of a 96-well plate (# 655090, Greiner Bio One) and left to air-dry under a biosafety cabinet. Coverslips were coated with 10 μg/ml Poly-D-Lysine solution (#A-003-E, Merck Millipore) in diethylpyrocarbonate (DEPC)-treated H_2_O and left under the biosafety cabinet overnight (50 μl/well). The next day, Poly-D-Lysine was removed and wells were washed three times with DEPC H_2_O to remove unbound Poly-D-Lysine. After air-drying, 10 μg/ml of laminin solution (# L2020, Sigma Aldrich) in sterile phosphate-buffered saline (PBS) was added to the wells (50 μl/well) and the plate was left at 37°C overnight. On the day of the dissection, laminin solution was removed and the wells were washed twice with sterile PBS and once with DEPC-treated H_2_O to remove unbound coating solution. After air-drying, 280 μl of Leibovitz’s L-15 Medium (# 11415-049, Gibco) supplemented with 15% fetal bovine serum (# 1000-0-044, Gibco, Performance Plus, Long Beach, CA, USA), 1% Glutamax (# 350-050-038, Gibco) and 1% penicillin/streptomycin (# 15-140-122, Gibco) was added to each well. The plate was left at 28°C until cell seeding. FACsorted OPCs were seeded at a density of 8,000 cells per well and left at 28°C until recording.

### Electrophysiology

Membrane currents were measured in the whole-cell configuration of the patch-clamp technique (Hamill et al., 1981; Wagner et al., 2014) at 3 days *in vitro* (DIV), using an EPC-9 amplifier (HEKA Elektronik) and the PATCHMASTER software (HEKA Elektronik). Pipettes were pulled from borosilicate glass capillaries (Science Products) and had resistances between 5 and 7 MΩ. Cell capacitance (*C*_m_) and series resistance (*R*_s_) were calculated using the automated procedure of the EPC-9 amplifier. *C*_m_ was 2.63 ± 0.10 pF and *R*_s_ measured 24.5 ± 2.4 MΩ (*n* = 101, *N* = 13).

Pipette potentials (*V*_Pip_) were corrected online for the liquid junction potential of 5.4 mV for K^+^ and Na^+^ channels and of 5.8 mV for glutamate receptor experiments. Leak currents were negligible and no correction was performed. Data were low-pass filtered at 5 kHz and sampled at 25 kHz for K^+^ and Na^+^ channels and low-passed filtered at 0.25 kHz and sampled at 1 kHz for glutamate receptor experiments. All experiments were performed at room temperature (22–25°C). Sustained current (I_mean_) was measured at the end of the voltage pulse (950–990 ms) and inactivating current component (I_A_) was quantified by subtracting the current at the end of the voltage pulse (I_mean_) from the peak current (I_peak_).

### Current- and Voltage-Clamp Protocols

For voltage-gated conductances, the cell membrane was clamped at a holding potential (HP) of −80 mV. For voltage-gated K^+^ channels, currents were measured in response to 1,000 ms voltage steps from −120 to +40 mV in 20 mV increments. Cycle length was 3 s. For voltage-gated Na^+^ channels, currents were measured in response to 500 ms voltage steps from −70 to +30 mV in 10 mV increments. Cycle length was 10 s. To elicit depolarizing spikes, increasing depolarizing currents of 500 ms duration starting from 10 to 70 pA were applied in the current-clamp mode. Cycle length was 10 s.

### Chemicals and Solutions

Salts were of analytical grade and obtained from Sigma-Aldrich. TTX was obtained from Carl Roth, Tetraethylammonium chloride (TEA) and 4-aminopyridine (4AP) from Sigma-Aldrich. AMPA, Cyclothiazide (CTZ), 2,3-dihydroxy-6-nitro-7-sulfamoyl-benzo[f]quinoxaline (NBQX), N-Methyl-D-aspartate (NMDA) and Glycine were obtained from Tocris Bioscience. For voltage-gated conductances, the basic extracellular solution contained (in mM): NaCl 137, KCl 2.9, CaCl_2_ 2.1, MgCl_2_ 1.2, HEPES 10, Glucose 10 (pH 7.8). The pipette solution was composed of (in mM): KCl 130, MgCl_2_ 4, EGTA 10, HEPES 10 and Na_2_-ATP 2 (pH 7.4). For glutamate receptors the basic extracellular solution contained (mM): NaCl 134, KCl 2.9, CaCl_2_ 2.1, MgCl_2_ 1.2, HEPES 10, Glucose 10 (pH 7.8). The pipette solution was composed of (in mM): CsCl 134, MgCl_2_ 2, EGTA 10, HEPES 10, Na_2_-ATP 4 and Li-GTP 0.4 (pH 7.4). During the measurements, solutions were applied by the DAD VM SuperFast perfusion system (Scientific Instruments) and driven away with the use of a CVC2000 vacuum pump.

### Data Analysis

Currents were measured using the FITMASTER software (Heka). All currents were normalized to cell capacitance and are given as current densities in pA × pF^−1^. *n* represents the number of experimental replicates (cells) and *N* represents the number of preparations. Microsoft Excel and GraphPad Prism were used to process the measured data. Data are presented as mean ± SEM. Significance was analyzed using an ordinary two-way ANOVA and a Tukey’s multiple comparisons test. ****P < 0.0001; ***P ≤ 0.001; **P ≤ 0.01; *P ≤ 0.05.

## Results

### RNA Sequencing Shows Differential Expression of Voltage- and Ligand-Gated Ion Channels Between OPCs and Oligodendrocytes

In mammals, progression through distinct stages of the oligodendroglial lineage is manifested by morphological differences and changes in membrane current properties (Gallo et al., 1996; Chen et al., 2008; Clarke et al., 2012). Thus far, however, membrane characteristics of OPCs from adult zebrafish have not been described.

**Figure 1:**
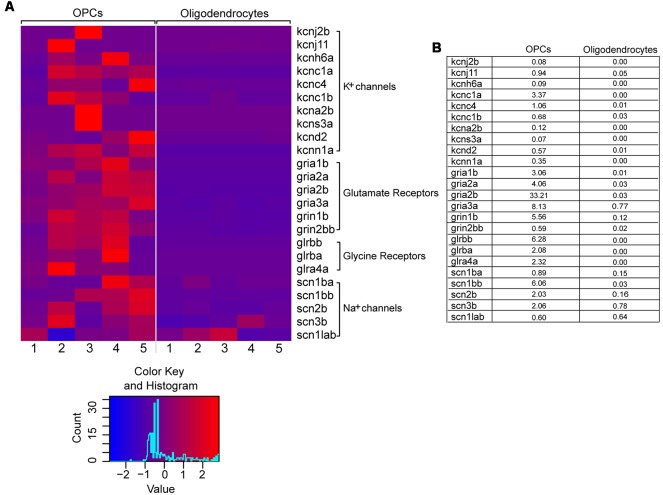
Differential gene expression of selected voltage- and ligand-gated ion channels in OPCs and oligodendrocytes. **(A)** Raw read counts are converted into transcripts per kilobase million (TPM) values, to make samples comparable. The expression levels are scaled by rows (per gene) to visualize differences in expression for each gene. The color key is showing the color scale for z-score distribution as well as a histogram about the distribution of scores. One to five refer to five biological replicates of transgenic Tg(*olig2*:eGFP) and Tg(*mbp*:eGFP) lines, respectively. **(B)** Mean expression values (in TPM) of OPCs and oligodendrocytes. On average the genes are relatively low expressed.

Therefore, we set out to examine the expression of cell-type specific ion channels in OPCs from the adult zebrafish spinal cord. To that aim, we used an RNA sequencing dataset previously published from our laboratory, in which the gene expression patterns of OPCs and oligodendrocytes from the adult zebrafish spinal cord were compared (Kroehne et al., 2017). We then filtered this dataset for genes that code for ion channels and receptors, based on their GO terms. Analysis on both cell types (OPCs and oligodendrocytes) revealed that adult OPCs from the zebrafish spinal cord differentially express, among others, a variety of voltage-gated K^+^ and Na^+^ channels as well as many neurotransmitter ligand-gated ion-channels, also described in mammals (Patneau et al., 1994; Chittajallu et al., 2004) (Figures 1A,B).

This suggests that membranes of adult zebrafish OPCs could exhibit ion current characteristics similar to mammalian OPCs.

### Adult Zebrafish OPCs Are Electrically Active

Based on the RNA sequencing data, we expected heterogenous functional expression of K^+^ conductances with sustained and inactivating outward rectifying properties, as well as expression of fast Na^+^ currents. Therefore, we further examined membrane excitability and current properties of purified OPCs by whole-cell patch-clamp. OPCs can be isolated from the adult zebrafish spinal cord in high purity and viability using the transgenic Tg(o*lig2*:eGFP) line, in which eGFP is driven by the *olig2* promoter, labeling OPCs (Kroehne et al., 2017).

**Figure 2:**
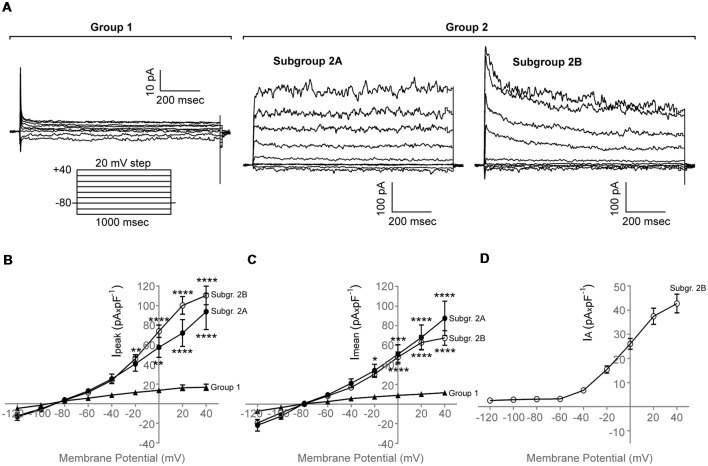
Whole-cell membrane current properties of adult zebrafish OPCs.** (A)** Representative whole-cell currents of three different cells from Group 1 and Group 2, respectively, in response to a voltage-step protocol that involved incremental application of 20 mV voltage steps from a holding potential (HP) of −80 mV (*n* = 65, *N* = 6). Group 2 consists of two subgroups, Subgroup 2A and Subgroup 2B. **(B,C)** Current-Voltage (I-V) plots of the peak (I_peak_) and sustained (I_mean_) current of the two cell groups observed (*n* = 10, 13 and 42, respectively, *N* = 6). I_mean_ was measured at the end of the voltage pulse (950–990 ms). **(D)** Current-Voltage (I-V) plot of the inactivating current component (I_A_) of Subgroup 2B. I_A_ was quantified by subtracting the current at the end of the voltage pulse from the peak current. Current amplitude was normalized to cell capacitance to correct for differences in cell size. Data are presented as mean ± SEM. Filled triangles: Group 1, filled circles: Subgroup 2A, open circles: Subgroup 2B. For means of clarity, significances are given as compared to Group 1. Significance was analyzed using an ordinary two-way ANOVA and a Tukey’s multiple comparisons test. ****P < 0.0001; ***P ≤ 0.001; **P ≤ 0.01; *P ≤ 0.05.

We found that purified adult zebrafish OPCs are electrically active (Figure 2A). Additionally, zebrafish OPCs fall into two distinct groups, defined by different degrees of outward rectification (*n* = 65, *N* = 6). In response to a depolarizing voltage-step protocol, the first group of cells (Figure 2A, Group 1) presents current conductances with lower degree of outward rectification and substantially lower amplitudes (I_mean_ at 40 mV, 11.3 ± 2.3 pA × pF^−1^, I_peak_ at 40 mV, 16.7 ± 3.2 pA × pF^−1^, *n* = 10; Figures 2B,C). Such low outward conductances have been previously described to correlate with differentiated stages of the oligodendroglial lineage (Sontheimer et al., 1989). In previous experiments, we did not detect MBP^+^ cells in our monocultures that would indicate the differentiation of OPCs into mature oligodendrocytes (Kroehne et al., 2017). As these cells could still, however, represent pre-myelinating oligodendrocytes, we focused for the rest of the study on the second group of cells (Group 2) that comprised of two subgroups with substantially higher outward conductances, as compared to Group 1, that activated at −40 mV (Figure 2A, Subgroup 2A and Subgroup 2B). Subgroup 2A presented large outward currents with sustained components (I_mean_ at 40 mV, 87.4 ± 17.6 pA × pF^−1^; I_peak_ at 40 mV, 93.9 ± 18.4 pA × pF^−1^, *n* = 13). Subgroup 2B presented also large outward currents (I_mean_ at 40 mV, 67.4 ± 7.5 pA × pF^−1^; I_peak_ at 40 mV, 110.4 ± 9.5 pA × pF^−1)^ with prominent transient, inactivating components (I_A_) (I_A_ at 40 mV, 42.3 ± 3.8 pA × pF^−1^, *n* = 42; Figures 2B–D).

### Adult Zebrafish OPCs Express Different Types of Functional Voltage-Gated K^+^ Channels

Outward rectifying conductances that are mediated through delayed rectifying voltage-gated K^+^ channels have been described in great detail in rodent OPCs. To test the functional expression of different voltage-gated K^+^ channels that were also identified in our RNA sequencing data, we performed the recordings in the presence of TEA and 4AP (Gutman et al., 2005).

**Figure 3:**
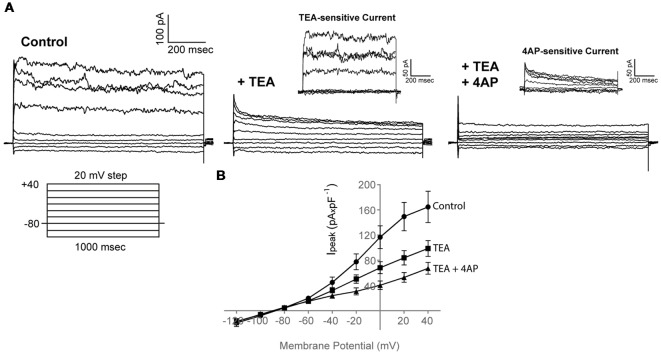
Adult zebrafish OPCs express functional voltage-gated K^+^ channels. **(A)** Representative whole-cell currents in response to a voltage-step protocol that involved incremental application of 20 mV voltage steps from a HP of −80 mV in basic extracellular solution (Control) containing 1 mM TTX and in the presence of TEA + 4AP (30 and 4 mM, respectively). Cells were constantly perfused with basic extracellular solution (Control) and at least for 50 s before recording with TEA and TEA + 4AP. TEA- and 4AP-sensitive currents are shown in insets and are derived from the currents shown in **(A)**. To isolate the TEA-sensitive current, the current traces recorded in the presence of TEA were subtracted from the traces recorded under Control conditions (I_peak_ of TEA-sensitive current at 40 mV, 54.4 ± 9.3 pA × pF^−1^, *n* = 19, *N* = 3). To isolate the 4AP-sensitive current, the current traces recorded in the presence of TEA + 4AP were subtracted from the traces recorded under TEA conditions (I_peak_ of 4AP-sensitive current at 40 mV, 42.1 ± 7.2 pA × pF^−1^, *n* = 19, *N* = 3). **(B)** Current-Voltage (I-V) plots of peak current (I_peak_) in Control, TEA and TEA + 4AP-containing extracellular solution (*n* = 19, *N* = 3). Peak current amplitude was normalized to cell capacitance to correct for differences in cell size. Data are presented as mean ± SEM. Filled circles: Control, filled squares: TEA, filled triangles: TEA + 4AP.

Application of 30 mM TEA resulted in a marked reduction of both the peak and sustained current component (I_peak_ at 40 mV in Control: 164.7 ± 24.8 pA × pF^−1^; I_peak_ at 40 mV in TEA: 98.8 ± 12.5 pA × pF^−1^, *n* = 19) that was potentiated further upon addition of 4 mM 4AP (I_peak_ at 40 mV in TEA + 4AP: 67.2 ± 9.4 pA × pF^−1^, *n* = 19), consistent with the presence of functional voltage-gated K^+^ channels, also present in our RNA sequencing data (like kcnc and kcnd) (Figures 3A,B, *n* = 19, *N* = 3).

Taken together, these results functionally confirm our RNA sequencing data and indicate that outward conductances in adult zebrafish OPCs are mediated through voltage-gated K^+^ channels.

### Adult Zebrafish OPCs Express Functional Voltage-Gated Na^+^ Channels and Exhibit TTX-Sensitive Spikes

In the adult rodent CNS, different classes of OPCs have been identified. These sub-populations have been shown to exhibit different electrical properties (spiking vs. non-spiking) and are reported to respond differently in cases of injury, representing potential subsets of cells with distinct functional properties (Káradóttir et al., 2008). Therefore, we tested whether zebrafish OPCs fall into marked subpopulations.

**Figure 4:**
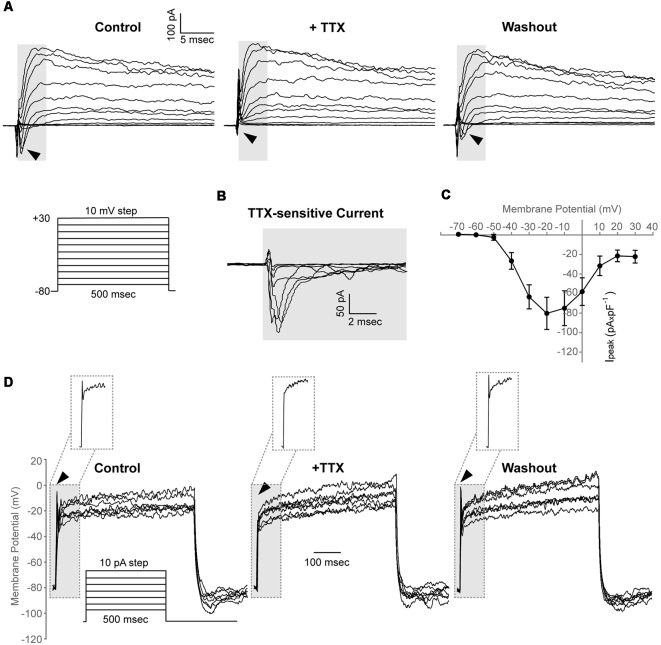
Adult zebrafish OPCs express functional voltage-gated Na^+^ channels and exhibit TTX-sensitive spikes. **(A)** Representative whole-cell currents in response to a voltage-step protocol that involved incremental application of 10 mV voltage-steps from a HP of −80 mV in basic extracellular solution (Control) and in the presence of TTX 1 μM. Cells were constantly perfused with basic extracellular solution (Control) and at least for 50 s before recording with 1 μM TTX. Highlighted background and black arrowheads indicate inwardly activating currents. **(B)** TTX-sensitive currents (highlighted background) are derived from the currents shown in **(A)**. To isolate the Na_v_ specific current, the current traces recorded in the presence of TTX were subtracted from the traces recorded under Control conditions. **(C)** Current-Voltage (I-V) plot of the peak current (I_peak_) of TTX-sensitive currents (*n* = 7, *N* = 4). Peak current amplitude was normalized to cell capacitance to correct for differences in cell size. Data are presented as mean ± SEM. **(D)** Representative whole-cell current-clamp recordings in response to depolarizing current injections of 10 pA. In basic extracellular solution (Control), current injections result in depolarizing spikes that are sensitive to 1 μM TTX and are restored after washout (highlighted background, black arrowheads and insets). *Insets*: magnification of the area within the highlighted dashed box.

We found that in 7 out of 30 cells (23%, *N* = 4), a depolarizing voltage-step protocol induced a rapidly activating transient inward current in voltage steps positive to −50 mV, that was blocked upon application of 1 μM TTX, indicating the functional expression of Na_v_ channels in adult zebrafish OPCs (I_peak_ at 0 mV, 58.1 ± 14.1 pA × pF^−1^; Figures 4A–C). Furthermore, in the cells exhibiting Na_v_-mediated currents, current injections gave rise to TTX-sensitive spikes that did not cross, however, 0 mV and were, therefore, not classified as action potentials (Figure 4D).

Taken together, these results suggest that adult zebrafish spinal cord OPCs comprise a heterogeneous population with similar electrical properties to rodent and hPSC-derived OPCs (Káradóttir et al., 2008; Livesey et al., 2016).

### Adult Zebrafish OPCs Express Functional Glutamate Receptors

Besides specific voltage-gated K^+^ and Na^+^ channels, based on our RNA sequencing data, adult zebrafish OPCs express a variety of ligand-gated ion channels. Among these, AMPA and NMDA receptors have been shown to be expressed in rodent OPCs and regulate not only their development but also post-injury responses (Bergles et al., 2000; Káradóttir et al., 2005; Fannon et al., 2015). Therefore, we further examined the functional expression of AMPA and NMDA receptors in our purified adult zebrafish OPCs.

**Figure 5:**
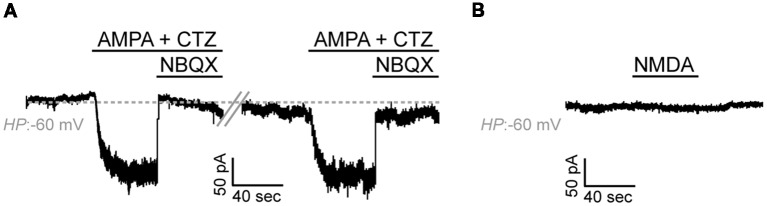
Adult zebrafish OPCs express functional AMPA receptors.** (A)** Representative, repetitive response of an OPC at a HP of −60 mV in basal conditions and during stimulation with AMPA 100 μM in the presence of the AMPA receptor desensitization inhibitor CTZ 50 μM. AMPA-mediated currents are blocked in the presence of the selective AMPA antagonist NBQX 150 μM. **(B)** Representative whole-cell recording in basal conditions and during application of NMDA 100 μM at a HP of −60 mV (in the presence of Glycine 50 μM).

Application of AMPA alone (100 μM) did not elicit detectable agonist-mediated currents which could be attributed to the low expression levels of AMPA receptors in our cells (data not shown). AMPA-mediated inward currents were, however, detectable upon application of the AMPA receptor desensitization inhibitor CTZ (50 μM) in 10 out of 17 cells (59%, Current Amplitude, 29.6 ± 8.2 pA × pF^−1^, *N* = 4; Figure 5A). Application of the selective and potent AMPA receptor antagonist NBQX (150 μM) blocked these currents. Application of NMDA (100 μM in the presence of Glycine 50 μM) did not elicit detectable currents in any of the cells recorded and in which AMPA-mediated currents were detected (*n* = 9, *N* = 3; Figure 5B).

Taken together, these results suggest that, similar to mammalian OPCs, adult zebrafish OPCs express functional glutamate receptors.

## Discussion

### Electrical Properties of Adult Zebrafish Spinal Cord OPCs

The distinct stages of OPC maturation are characterized by the differential expression of a variety of ligand-receptor pairs and ion channels (Sontheimer et al., 1989; De Biase et al., 2010) that not only orchestrate the OPC-specific responses during lineage progression but also upon activity-dependent myelination (Gautier et al., 2015), injury and remyelination (Káradóttir et al., 2005). It is by now clear that the OPC membrane properties observed in rodent systems, exhibit strong conservation with hPSC-derived OPCs (Livesey et al., 2016). However, the development of successful remyelination therapies may profit from a thorough knowledge of OPC properties also in regenerating species.

### Voltage-Gated Channel Expression in Adult Zebrafish OPCs

Using an *in vitro* platform developed in our laboratory (Kroehne et al., 2017), this study addressed whether OPCs from the adult zebrafish spinal cord exhibit similar and/or distinct membrane current characteristics as compared to mammalian OPCs. Data acquired in this study provide the first functional evidence that similar to their mammalian counterparts, OPCs from the adult zebrafish spinal cord are electrically active. They express functional voltage-gated K^+^ channels with degrees of outward rectification previously described in rodent (Barres et al., 1990) or embryonic stem cell-derived OPCs (Husseini et al., 2008; Jiang et al., 2013). Additionally, adult zebrafish OPCs exhibited small inward conductances, indicative of inwardly rectifying K^+^ channels. Such small currents, could be attributed to the isolation and culture conditions that potentially affect OPC-processes, in which these channels are primarily localized, and that have been previously shown to result in reduced or even absent inward currents in mammalian OPCs (Newman, 1984; Brew et al., 1986; Barres et al., 1988). Moreover, in mammals, inwardly rectifying K^+^ channels are significantly upregulated as OPCs mature into oligodendrocytes, where they also hold key roles in regulating myelination and extracellular K^+^ accumulation (Sontheimer et al., 1989; Neusch et al., 2001; Maldonado et al., 2013).

Taken together, such data from mammalian OPCs have by now shown that voltage-gated K^+^ channels exert key roles in orchestrating cell cycle progression of OPCs while regulating tissue homeostasis (Knutson et al., 1997; Ghiani et al., 1999). The data obtained in this study suggest that ion channel expression during zebrafish oligodendrogenesis, may correlate with the shifts in membrane conductances observed in mammals.

Furthermore, it was demonstrated that a subset of zebrafish OPCs exhibits TTX-sensitive spikes mediated through functional Na_v_ channels. However, due to their low amplitude that did not cross 0 mV, these spikes cannot be classified as *bona fide* action potentials that were also reported to be absent in postnatal murine OPCs of the gray and white matter (De Biase et al., 2010) and were, therefore, characterized as depolarizing spikes. This is, however, in contrast to data from hPSC-derived OPCs and OPCs in the rodent CNS white matter that were previously shown to exhibit regenerative action potential firing in response to depolarization (Káradóttir et al., 2008; Stacpoole et al., 2013). It could be argued that the culture conditions used in this study prompted OPCs towards differentiation and, therefore, induced the downregulation of Na_v_-mediated currents and the firing of action potentials that has been shown to be restricted in undifferentiated progenitors (Barres et al., 1988). Nevertheless, previous characterization of the cultures makes such a scenario improbable (Kroehne et al., 2017). Interestingly, we observed that the percentage of zebrafish OPCs exhibiting TTX-sensitive spikes and Na_v_-mediated currents was markedly lower compared to rodent OPCs, where the class of action-potential-generating OPCs are preferentially vulnerable to excitotoxic damage due to the higher expression of glutamate receptors (Káradóttir et al., 2008). In this context, it remains to be studied how the membrane current properties of zebrafish OPCs instruct their injury responses and potential injury susceptibility.

### Glutamatergic Signaling in Purified Adult Zebrafish OPCs

Next, we investigated the functional expression of glutamate receptors, previously described to be expressed in mammalian OPCs (Berger et al., 1992; Káradóttir et al., 2005), controlling their proliferation potential, migration and, therefore, development and differentiation (Wang et al., 1996; Yuan et al., 1998; Gudz et al., 2006). We showed that adult zebrafish OPCs express functional AMPA receptors but no NMDA-mediated currents were detected. This is in accordance with previous experiments, in which blocking of AMPA but not NMDA receptors perturbed the morphological development and lineage progression of mammalian OPCs, suggesting that the activity-dependent signals orchestrating OPC biology are actually mediated through AMPA and not NMDA receptors (De Biase et al., 2011; Fannon et al., 2015). As rodent OPCs have been shown to express NMDA receptors and low NMDA currents also activate upon injury (Káradóttir et al., 2005), another possibility for the absence of detectable NMDA-currents could also be the short culturing period before the acquiring of the recordings and the absence of synaptic connections in such an *in vitro* preparation. This has been previously described to down-regulate the functional expression of NMDA receptors (Káradóttir and Attwell, 2007) that were also absent in cultured OPCs (Patneau et al., 1994; Livesey et al., 2016). Along these lines, it has been also shown that different neuronal-activity-triggered stimuli differentially affect OPC current responses (Nagy et al., 2017). This could again highlight a potential limitation of the *in vitro* setup used, in detecting ionic events depending on axon-OPC synaptic input.

Taken together, the electrophysiological data obtained in this study demonstrate that the membrane properties of OPCs from the adult zebrafish spinal cord are highly similar to mammalian OPCs. Given the implication of both voltage-gated channels as well as AMPA and NMDA receptor activation in glutamate-mediated excitotoxicity and demyelination in mammals (Wosik et al., 2004; Micu et al., 2006; Song et al., 2018), it would be of particular interest to examine how OPC membrane current properties change upon injury and/or during the process of (re)myelination in a regenerative species. The *in vitro* platform proposed in this study, could be utilized towards that aim.

The identification of signaling components orchestrating OPC injury responses in a highly competent regenerative context could provide novel insight towards successful remyelination strategies in non-regenerating species.

## Author Contributions

VT, MB, MW and MR: conceptualization. VT, VK and SR: investigation. VT and MR: writing. AE-A, MB and MR: resources.

## Conflict of Interest Statement

The authors declare that the research was conducted in the absence of any commercial or financial relationships that could be construed as a potential conflict of interest.
